# Effect of betulin oil on hair growth in hypothyroidism- a long-term blinded pilot study

**DOI:** 10.1007/s00403-024-02913-5

**Published:** 2024-06-01

**Authors:** Agata Serrafi, Karolina Gostyńska, Adrian Kasprzak, Andrzej Wasilewski, Piotr Marczyński, Sylwiusz Kontek, Wojciech Lewandowski

**Affiliations:** 1 Department of Immunochemistry and Chemistry, Wrocław Medical University, M. Skłodowskiej-Curie Street 48/50, 50-369 Wrocław, Poland; 2 Faculty of Prevention and Health, Medical College Ul. Nowowiejska, 69 50-340 Wrocław, Poland; 3 Student Scientific Association of Medical Chemistry and Immunochemistry, Wroclaw Medical University, 50-369 Wroclaw, Poland; 4 Faculty of Medicine, Wrocław Medical University, 50-367 Wrocław, Poland

**Keywords:** Betulin, Hypothyroidism, Betula, Hair loss, Pilot study

## Abstract

**Background:**

One common problem in various patient groups is excessive hair loss on the head. One such group is people struggling with hypothyroidism. The market for preparations for hair growth and hair loss prevention includes betulin.

**Purpose:**

This pilot study investigated its effect on hair loss in hypothyroid patients.

**Study design:**

The study included a group of hypothyroid patients and a control group of people without hypothyroidism. Participants were randomly divided into a group taking placebo and betulin.

**Methods:**

Results were investigated using photographic assessment of hair, trichoscopy and subjective evaluation of participants.

**Conclusion:**

The study did not conclusively prove that betulin would contribute to the inhibition of hair loss or regrowth.

**Graphical Abstract:**

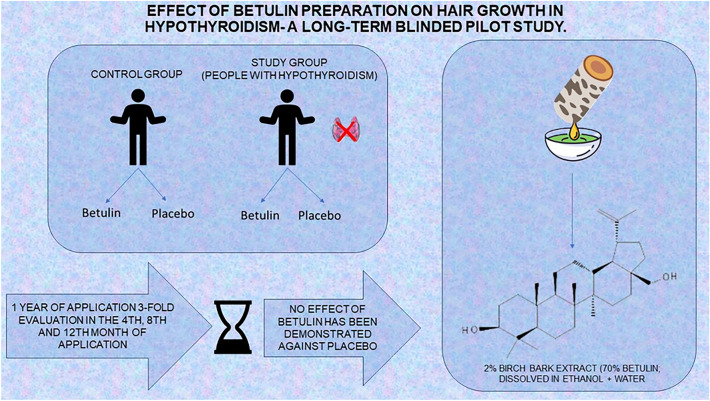

## Introduction

**Fig. 1:**
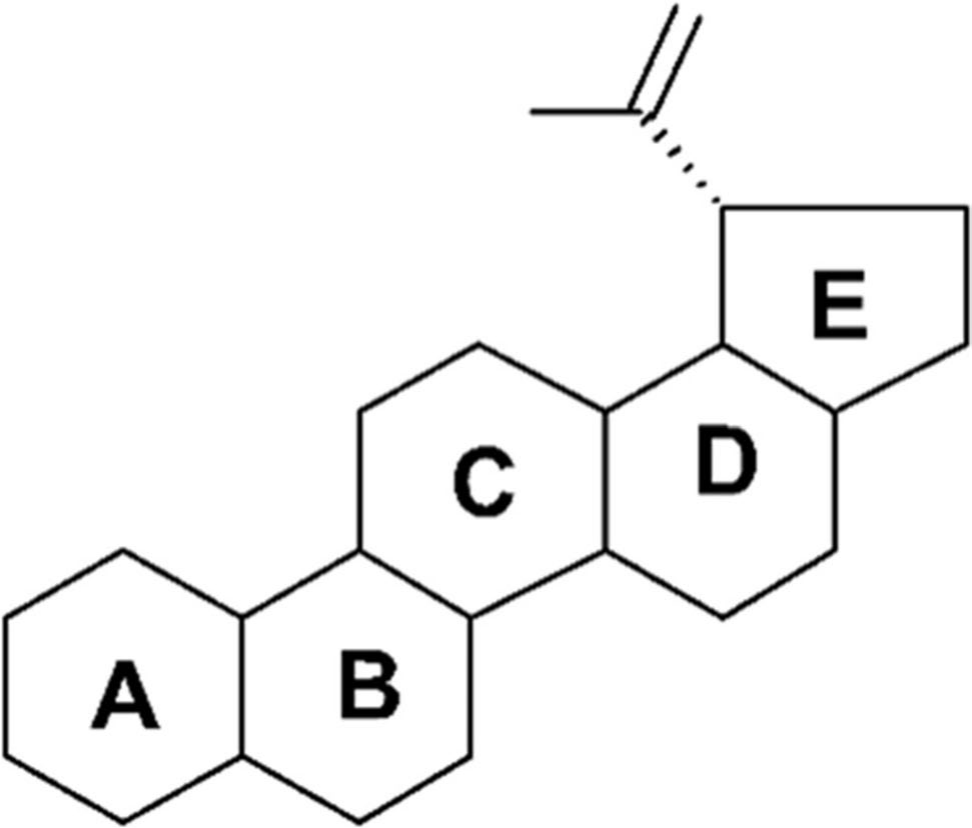
Chemical structure of the lupane skeleton

Betulin and betulinic acid, (3 β-hydroxy-lup-20(29)-ene-28-oic acid) (Fig. 1) are naturally occurring pentacyclic lupane—a type of triterpenoid that exhibit biodiversity and medicinal properties such as inhibition of viral immunodeficiency virus (HIV) [1, 2], antibacterial [2, 3], antimalarial [4], anti-inflammatory [5–8], anthelmintic [9], antinociceptive [10], anti-HSV-1 [11, 12] and anticancer [13–17]. Birch (*Betula spp.,* Betulaceae) is one of the most widely transferred sources of betulinic acid and betulin, which can be obtained in significant amounts [18–20]. Betulinic acid can also be isolated from various sources including *Ziziphus spp.* (Rhamnaceae) [16, 21], *Syzygium spp.* (Mojrtaceae) [1, 22], *Diospyros spp.* (Ebenaceae) [23–25] and *Paeonia spp.* (Paeoniaceae) [26]. The reduced congener of betulinic acid, betulin (3 β-lup-20(29)-ene-3,28-diol), was one of the first natural products isolated in 1788 from the bark of the white birch Betula alba [27].

Thyroid hormones play an important role in the proper functioning and development of the body, starting from the stage of embryogenesis. They affect, among others, the development and function of the brain and nervous system, metabolism of proteins, carbohydrates and fats, the development of kidneys and the skeletal system, calcium and phosphate metabolism, heat production in the body and the condition of the skin or hair. Hashimoto’s chronic lymphocytic thyroiditis is an autoimmune disease. The aim of the article was to draw attention to the current aspects related to the etiopathogenesis and side effects associated with Hashimoto’s disease, which affect the process of baldness [25–27].

This article focuses on the effects of betulin oil on hair growth in people with hypothyroidism.

### The use of betulin in cosmetology

The interest of cosmetologists is aroused by birch bark as a source of natural triterpenoids. The results of studies described in the literature clearly indicate the legitimacy and wide range of possibilities of using this raw material and compounds isolated from it in cosmetics: betulin, betulinic acid and lupeol [2, 19–32]. Betulin, by inhibiting the secretion of histamine, has anti-allergic properties [2]. It is used in preparations intended for the care of sensitive skin, prone to allergies. It also seems reasonable to introduce betulin into formulas of cosmetic preparations for mature or aging skin, as it exhibits antioxidant properties related to the inhibition of superoxide radical production [29–33]. By preventing oxidative stress, it has a protective effect on the skin and slows down the aging process. It also has an inhibitory effect on the herpes simplex virus, accelerates wound healing and reduces swelling. The need to apply betulin to the epidermis prior to procedures that compromise the continuity of the epidermis is confirmed by, among others, study by Metelmann et al., who showed that after skin resurfacing procedures using a CO_2_ laser, aesthetics were better after using an emulsion containing betulin than using a hydrocolloid dressing [30]. In Italy, a paste made of birch bark is used as a poultice for cuts, wounds and burns, and a decoction of birch bark for ulcers. In Bosnia and Herzegovina, a liquid containing the bark and leaves of the silver birch (Betula pendula Roth) is used to stimulate hair growth and eliminate dandruff. In Southeastern Europe, birch bark is used in many skin diseases, and in Italy, birch decoction is also used to wash and rinse hair, as a means of preventing hair loss [34–36].

### Effects of thyroid hormonal disorders in scalp and hair

**Fig. 2:**
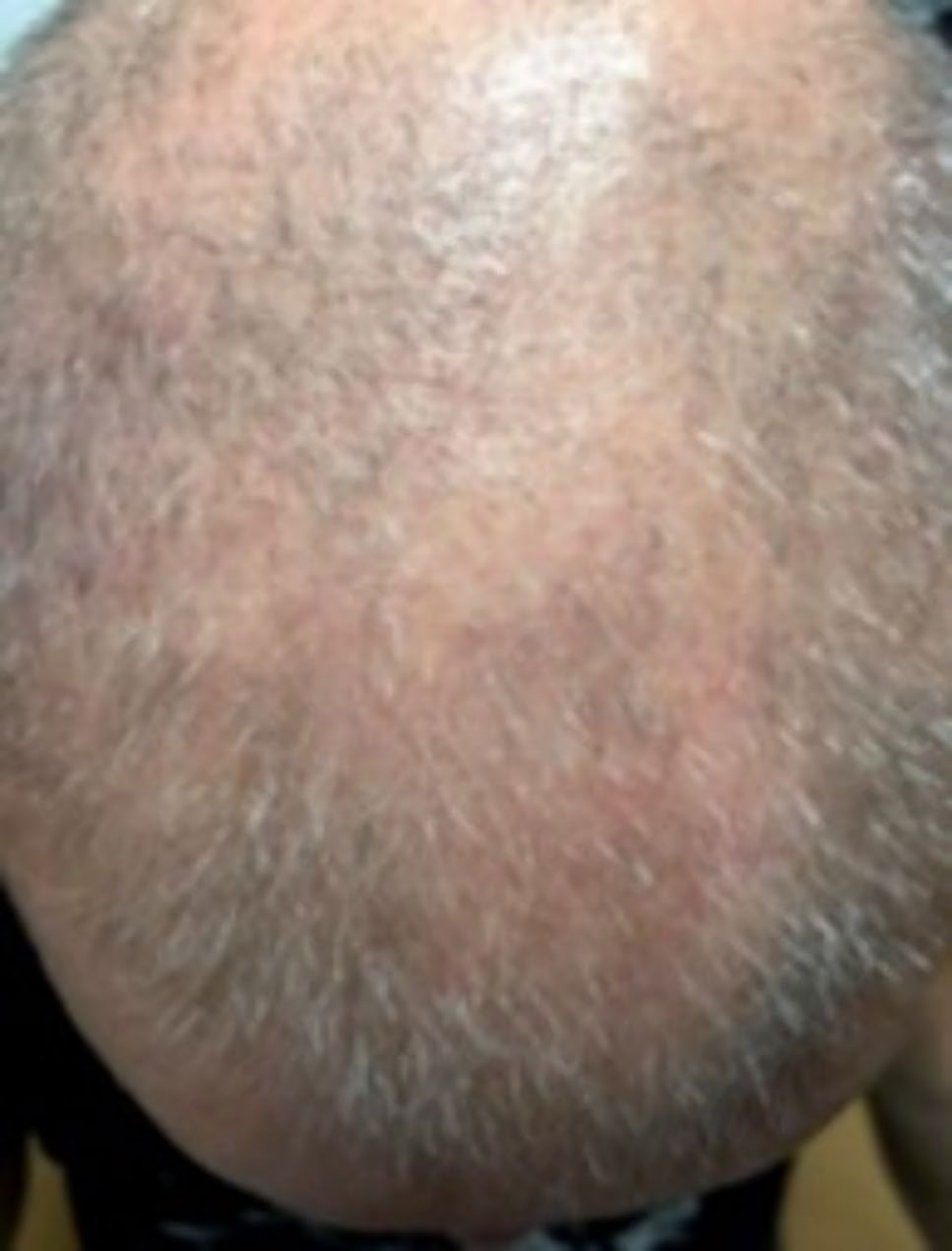
Scalp with areas of visibly less hair density in a patient with Hashimoto’s disease and autoimmune diseases. Authors’ own archive

During the symptomatic phase of hypothyroidism in Hashimoto’s disease, pathological changes occur within the skin and its appendages. The skin is cold and pale due to vasoconstriction and reduced blood flow, with a yellowish tint, showing features of excessively horny epidermis, especially in the area of elbows and knees. The skin becomes dry, rough and excessively flaky [37]. It has been shown that there is a relationship between the occurrence of alopecia and the presence of antibodies directed against the thyroid gland, i.e. anti-TPO and anti-Tg, but no significant correlation was observed between hormonal disorders of the thyroid gland and the frequency of alopecia [38]. This suggests that the main factor responsible for the process of alopecia in autoimmune diseases (including Hashimoto’s disease) are autoimmune processes (associated with the presence of elevated concentrations of antibodies), but not related to abnormalities in the clinical picture or thyroid hormone levels [38]. The common mechanism of alopecia lies in the skin, and we can look for causes from disorders of the sebaceous and sweat glands and disorders of keratin metabolism. Subcutaneous oedema may occur. myxoedema, mainly in the vicinity of a significant accumulation of flaccid connective tissue, which can lead to thickened facial features, swelling of the eyelids and hands due to the deposition of fibronectin and hydrophilic glycosaminoglycans in the subcutaneous tissue, the synthesis of which is inhibited by trio to thyronine. Hair is dull, thinner, brittle and may fall out eyebrows (Fig. 2) [39, 40].

In a number of clinical studies after searching for an association between alopecia and immunological diseases, a meta-analysis of these studies showed that there is a significant co association between the occurrence of alopecia, androgenetic alopecia, which may also occur among patients with Hashimoto’s disease [37, 41, 42]. Patients suffering from Hashimoto’s disease struggle with many unpleasant ailments. For many patients, this is very burdensome, hence seeking help both from a doctor and on one’s own. Some of the symptoms can be counteracted to some extent by making the diagnosis quickly enough and implementing appropriate treatment. Also at home, with the right diet, skincare or medical treatments, the side effects of Hashimoto’s disease manifesting on the scalp can be alleviated.

### Care options

In the case of Hashimoto’s disease, the care aspect can be considered, which can give satisfactory results when combined with appropriate treatment and diet. In the case of problems with baldness, we can still stimulate the hair follicles from the outside. One way is to inhibit the enzyme 5α-reductase, which can be influenced by water-alcoholic extracts of saw palmetto, green tea, pumpkin seeds or liquorice. It is also important to combine individual extracts, as it has been proven on the example of pumpkin seeds and saw palmetto that they work better when mixed than when administered separately. Alcohol-based solutions with warming additives such as rosemary or chili pepper extracts improve blood flow and become a transition promoter for extracts. Substances that improve the proliferation and activity of hair follicles, for example, grape seed oil applied to the scalp before washing, may have an equally good effect [43].

It is also recommended to use birch extract in the care and therapy of skin diseases, as well as in the case of excessive hair loss, leading to baldness [43, 44]. Proper hair care and nutrition of the body and the introduction of appropriate supplementation will supplement eliminating deficiencies, which are necessary to inhibit the process of baldness and proper hair growth. Few studies indicate specific care options for people affected by alopecia. All of the listed additives introduced during the care may, but do not have to, improve the condition of the scalp.

The aim of the project is to investigate whether betulin oil affects hair loss in patients with hypothyroidism. The current pilot study is intended to provide initial predictions and also to identify advice for other researchers on what elements to look out for in further studies on this topic.

## Materials and methods

### Study design

**Fig. 3:**
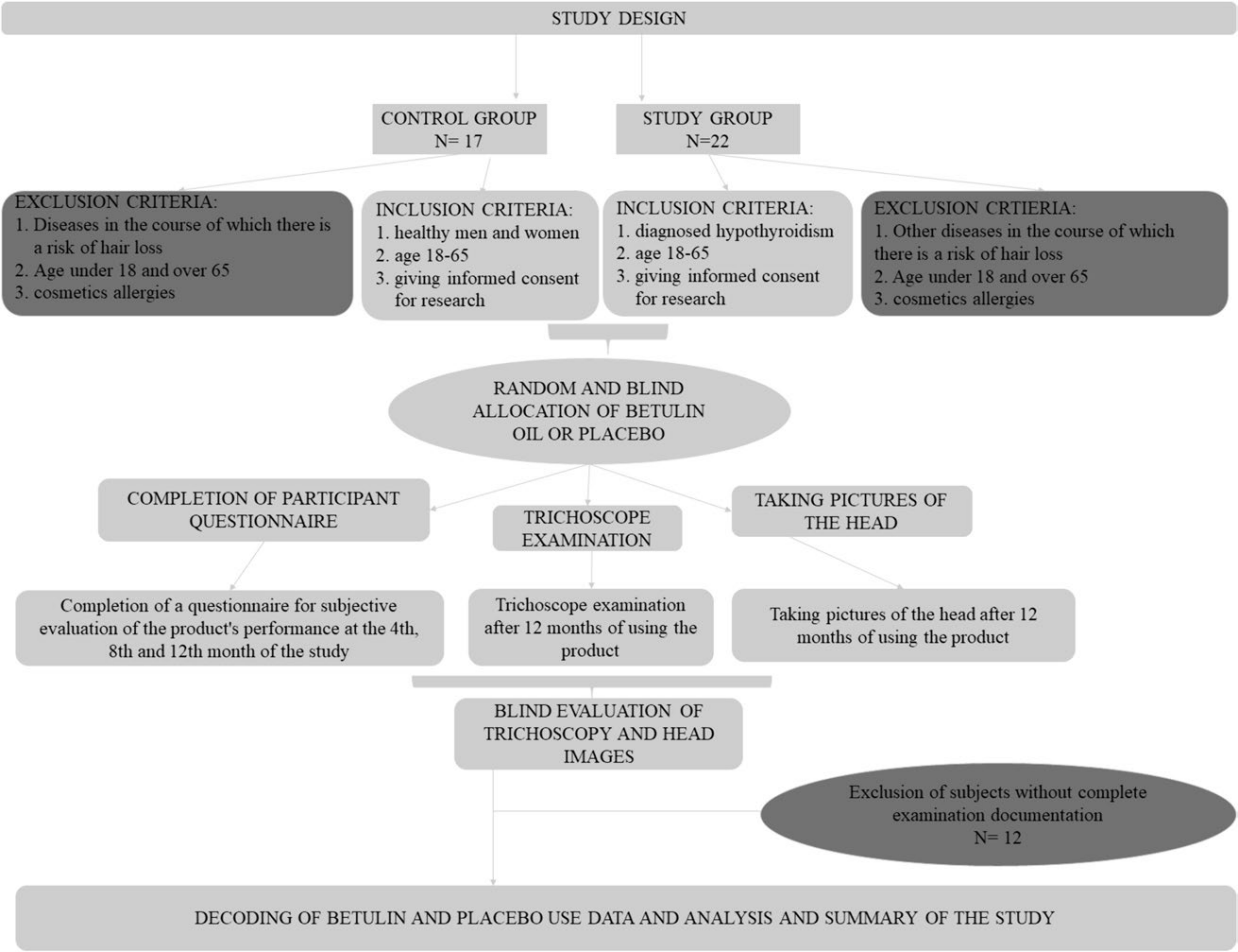
Scheme for conducting the survey

The study lasted from 1.10.2021 to 30.03.2022. People from the research team’s local community were invited to participate in the study. Approval for the study was obtained from NWSM/1/09/2021. Candidates for the study were selected with the aim of being as similar as possible in the group of healthy and hypothyroid individuals in terms of sex and age, as well as the hair loss problem. The subjects selected for the study are shown in Table 1. Each potential candidate for the study was personally informed about the study’s conduct and method, as well as the subsequent use of the data. Each respondent gave written voluntary and informed consent to participate in the study. Ultimately, 39 subjects participated in the study, and the study design including inclusion and exclusion criteria is shown in Fig. 3. Prior to the start of the study, subjects completed a medical and community interview questionnaire, including questions about: diagnosis of thyroid disorder (duration of illness, method of treatment, being under the care of a specialist, symptoms of illness), basic data (age, gender, place of residence, education), comorbidities, hair loss problems (degree of hair loss, previous treatment attempts and care). Participants had the opportunity to constantly interact with the research team and consult any concerns. They were informed that they could drop out of the study at any time and that if they experienced any worrisome symptoms, they should immediately stop taking the product, contact the research team and their doctor. Subjects from the control group (healthy subjects) and the study group (hypothyroid subjects) were randomly allocated to the placebo group and the betulin oil group. The subjects were not informed whether they were taking betulin oil or placebo, but were informed that the study was with the placebo group and they could be placed in the placebo group. The part of the research team responsible for contacting the subjects and dispensing the formulation also did not know which formulation they were dispensing, while the part of the team responsible for coding the data and samples had no contact with the subjects. The endpoint of the study is the attainment of a minimum of one unit higher on the Hamilton or Embling Rook scale and/or the appearance of trichoscopic changes indicating intensification of hair growth.

**Table 1:** Demographic data

	Healthy persons *n* = 11	People with hypothyroidism *n* = 16
Age		
18–26	10 (90.9%)	9 (56.3%)
27–35	0	1 (6.4%)
36–50	1 (9.1%)	4 (25%)
> 50	0	2 (12.5%)
Gender Male/female	3 (27.3%)/ 8 (72.7%)	2 (12.5%)/ 14 (87.5%)
Placebo/betulin	4 (36.4%)/ 7 (63.6%)	5 (31.3%)/ 11 (68.7%)

### Composition of the study preparation and placebo. Method of application

The test oil was a suspension of 2% birch bark extract, containing approximately 70% betulin; approximately 40% ethanol and water. The placebo preparation was prepared with 40% ethyl alcohol and water. The formulation was applied to the washed scalp, once daily in the evening diluted with boiled water at a ratio of 1:10. This was repeated daily throughout the study.

### Blinding procedure

The blinding procedure was as follows:

- 1. Allocation to the placebo and test groups was drawn, and the test subject was given a preparation in the same package, colour and odor regardless of group allocation.
- 2. Other people from the research team were assigned to dispense the preparations and others to interpret the results.
- 3.After the study data was collected, trichoscopic evaluation, photos and interpretation of subjective assessment were performed before data was decoded on a person’s membership in the control or study group.

### Periodic checkups

After 4, 8 and 12 months of the study, participants completed a questionnaire on their subjective evaluation of the results. Participants were informed at what time they would be asked to complete the follow-up questionnaire. In each of the given months of the study, the team members responsible for contacting participants by telephone reminded them to complete the survey, which was sent electronically. They answered questions about perceived increased or decreased hair loss, hair condition, side effects, reactions and scalp condition. The questionnaires were collected from the participants online using Google Forms, the tool recorded the dates when the questionnaire was completed and failure to complete the questionnaire, incomplete completion or delay of more than seven days resulted in exclusion from the study. Responses from the surveys were analysed and collated in the results section.

### Analysis of trichoscopy and photos

Macroscopic observation of the scalp and trichoscopy using a Levenhuk DTX 90 camera, which allows 300 × magnification, were used to analyse the effect of the preparations used. To analyse the collected data, in the case of men, the Hamilton scale was used, which is based on the analysis of frontal and parietal recessions and frontal thinning. This scale assumes two categories of hair loss—scalp that is not bald (types I-III) and scalp that is bald (types IV-VIII) The scale is one of the most popular in the diagnosis of male pattern baldness, and is the prototype for other diagnostic scales (**1**). In the case of women, the five-stage Ebling and Rook scale (I-V) was used, which is an expanded version of Ludwig’s classification (**1**, **2**). When these scales were used, the effect before and after therapy was tabulated. The trichoscopy used was twofold—in order to exclude dermatological diseases of the scalp, Peripolar halo was looked for, which could indicate peri folliculitis, blue—grey dots could indicate lichen, hyperkeratotic suppositories could indicate lupus (**3**). Secondly, trichoscopy also looked for the appearance of so-called ‘baby hair’ which is a good measure of new hair growth (**4**). The treated patients were classified ± in terms of the appearance or absence of ‘baby hair’.

### Statistical analysisxz

The collected data were processed using Microsoft Office Excel and Statistica 13.3 (Wroclaw Medical University’s license, StatSoft Polska Sp. z o.o.). Due to the handling of small data, the Yates continuity correction was applied, and the Chi-square test of independence was used to assess the significance of the data obtained.

### 7. Sample size calculation

The sample size was calculated on the basis of data from the Central Statistical Office of Poland [45], and is 385 with a confidence interval of 96% and a margin of error of 5%.

## Results

### Demographic data

Thirty-nine subjects were included in the study, with 17 subjects in the control group (healthy subjects) and 22 subjects in the study group (hypothyroid). After verification of the collected data, the results of 12 subjects were excluded (Fig. 3).

All patients with hypothyroidism are under the care of an endocrinologist and are taking levothyroxine. During the course of the study, their condition was stable with no significant changes in the course of the disease.

### Does betulin work?

Changes over 12 months were divided into subjective and objective, where objective includes trichoscopy assessment and head radiographs. Due to the small group of subjects, the results were divided in a binary way—into a group where there was an improvement and a group where there was no improvement. The data obtained through trichoscopic assessment, headshots and subjective assessment are summarised in the tables (Tables 2, 3, 4) below. No statistically significant changes were observed in any of the listed groups.

**Table 2:** Trichoscopic evaluation

Betulin preparation			
	Improving	No Improvement	*p*-Value
People with hypothyroidism	10	1	0.811
Healthy persons	7	1	
Placebo			
	Improving	No Improvement	*p*-Value
People with hypothyroidism	4	1	0.783
Healthy persons	3	0

**Table 3:** Assessment of headshots

Betulin preparation			
	Improving	No Improvement	*p*-Value
People with hypothyroidism	10	1	0.353
Healthy persons	5	3	
Placebo			
	Improving	No Improvement	*p*-Value
People with hypothyroidism	2	3	0.999
Healthy persons	2	1

**Table 4:** Subjective evaluation

Betulin preparation			
	Improving	No Improvement	*p*-Value
People with hypothyroidism	5	6	0.788
Healthy persons	4	4	
Placebo			
	Improving	No Improvement	*p*-Value
People with hypothyroidism	1	4	0.144
Healthy persons	3	0	

### Does betulin work in patients with thyroid conditions?

**Table:** 

Trichoscopic evaluation			
	Improving	No Improvement	*p*-Value
Betulin preparation	17	2	0.602
Placebo	7	1	
Assessment of headshots			
	Improving	No Improvement	*p*-Value
Betulin preparation	15	4	0.297
Placebo	4	4	

### Other results

The other effects that the respondents were asked about concerned the impact of the product on the scalp and hair condition. All the effects observed were transient and had no significant impact on the study’s safety or the reliability of the data.

In one healthy subject taking the placebo, the scalp was found to be less dry. A similar change was observed in the group taking the betulin preparation, with four subjects experiencing this effect. Among these four subjects, two had hypothyroidism, and the other two were healthy.

Moreover, increased dryness of the skin was noticed in four subjects within the betulin oil group. Similar to the previous case, this change was observed in two healthy subjects and two subjects with hypothyroidism.

Additionally, one subject in the betulin-using group with hypothyroidism showed a reduction in scalp flaking, while a healthy subject in the same group experienced increased scalp flaking.

## Discussion

Betulin is a substance of natural origin from the triterpene group, a pentacyclic diol found primarily in birch bark. Numerous studies report its anti-inflammatory and anti-HIV activity, as well as its positive effect on wound healing [12]. Among the literature, its anticancer activity is also widely demonstrated [13]. One of betulin’s actions may also be an effect on hair growth, which is used in several natural methods deriving this substance from plants. The problem of hair loss also has far-reaching consequences, such as a deterioration in self-esteem and well-being, the prevention of which is particularly important in the context of the rising trend in the incidence of mental illness in recent years. One natural method widely used in many parts of the world, such as the Pacific region or Polynesia, is to treat the hair with oils or extracts of selected plants in their composition among other things that contain betulin [4, 5, 7, 9, 10]. Oils and extracts from these plants are widely recognised as effective means of improving hair growth and slowing hair loss. The high popularity of these natural methods is due to their long-term use and the positive results they provide. Regular use of oils and plant extracts can also help to prevent hair breaking and splitting. Natural hair care methods using oils and plant extracts are often favoured by those looking for alternative, organic hair care products. Many people believe that natural methods are less damaging to the hair and scalp than chemical products, which contributes to their popularity. Although a significant amount of scientific evidence denies the effects of betulin on hair growth, many people around the world continue to use oils and extracts of plants containing it for hair care due to their traditional use and positive reviews. Plants used for this purpose include *Calophyllum inophyllum* (flowers and leaves) and *Schleichera oleosa* (bark), from which local people have extracted hair-improving substances for generations. When rubbed into the skin, preparations made from these plants increase hair growth. The action of betulin also includes cleansing the hair, which improves its condition and growth [11]. The use of betulin-containing preparations in order to stop hair loss has been patented in the United States [3].

Among the studies conducted on betulin, there are also many which prove that it has no significant effect on hair growth, which also corresponds to our observation. In our study there was no clinicallly significant effect of birch bark extract containing betulin on the hair growth of the patients. When evaluating the effect of isolated betulin preparations, the expected effects observed when using the above-mentioned natural methods were not achieved. Studies indicate that betulin has a beneficial effect on wound healing, but the issue of new hair growth remains unclear [1, 2, 6].

However, it is important to note that research is still ongoing and scientists are trying to explore the effects of betulin-containing products and their potential properties. Further research may result in a clearer determination of the action of betulin preparations in terms of hair growth. Nevertheless, on the basis of current scientific knowledge, it cannot be concluded that betulin is significant for hair growth. These data contradict the practice of using various oils and natural preparations, but they do not prejudge the effect or lack of effect of betulin on hair growth. In order to determine the effect of betulin, an extended cohort study should be conducted [6, 16].

The direct effect of betulin on the studied aspects of human health has not been demonstrated with such accuracy, probably due to the use of ethanol alcohol solution as a placebo trial, in which a large number of positive results were also observed. Ethyl alcohol is used in broad-spectrum medicinal products [16]. The concentration of 40% ethyl alcohol in the solution used here will have very weak bactericidal and fungicidal properties, because it has been shown that ethyl alcohol has the best antifungal properties in solutions of at least 70 percent [17], so the improvement in hair condition after placebo could not be due to the antiseptic properties of ethanol. In chemistry, ethyl alcohol is described as an excellent solvent of hydrophobic compounds [18], and when applied apically to the skin, it will cleanse excess biological material by removing dead epidermis and causing apoptosis of older epidermal cells [18] and dilating blood vessels [16]. These properties increase the effect of active substances in preparations applied to the scalp by increasing their solubility in the case of hydrophobic compounds [19]. In the case of a research trial using betulin in the preparation, ethyl alcohol could increase its activity, but using it in a control trial as a placebo could increase the effect of hygiene products used on a daily basis by the group of test subjects, which is why the control group could have had a greater amount of people with improvement of the examined situation. However, not all studies indicate that ethyl alcohol is an ideal solvent in medical or hygiene products, and there are also numerous studies indicating the negative effects of using ethanol-based preparations. Depending on percentage of ethanol in solution and place of usage side effect could be different [21]. Ethanol contributes to excessive drying of the skin [16], which could have caused this condition in the above-mentioned subjects. Likewise, excessive flaking of the scalp could also be caused by the increased amount of application of ethanol preparations to the scalp [16]. The use of ethanol could have had a significant impact on the results published in the study, therefore, in subsequent research work, an attempt could be made to use a pure extract of betulin or another organic solvent [16]. However, research on the effect of ethanol on the skin is still a broad topic discussed by scientists [21, 22]. It contributes to delayed wound healing and the occurrence of many skin diseases, while when consumed it may contribute to an increased risk of melanoma [21].

A limitation of the study was the lack of determination of T3 and T4 levels; these parameters should be included in future studies. The literature on the action of betulin, and particularly on the action of betulin in people with conditions manifesting as alopecia, is very scarce, demonstrating the need to expand this topic in future studies. When conducting further research, it is advisable to properly educate the subjects about the need for appropriate application of ethanol-based preparations or to refrain from using ethanol due to the possibility of causing skin keratosis, peeling, skin deodorization and many others. When analyzing research results, the placebo effect should also be considered, which may affect the research results [23], in particular the subjective assessment of health improvement among the study group. The study participants were not informed whether they were receiving a placebo or a oil containing betulin, which may result in an increased search for effects where they do not necessarily have to be present [24].

## Conclusions

The data obtained in the pilot study conducted by our team did not show any significant change in the effect of betulin compared to placebo. However, due to the small size of the study group, this does not allow firm conclusions to be drawn about the effect, or lack thereof, of betulin use on hair growth and further studies are needed to establish this. The project of subsequent research proposed:

- Changing the form of placebo.
- Use of pure betulin preparation.
- Expanding the interview with accurate laboratory determinations of thyroid hormone concentrations.

Future research should aim for the number of respondents resulting from the sample size.

Properly performed scalp care treatments can improve its condition and thus alleviate hair symptoms related to the disease process.

## Data Availability

Data and materials are available from the correspondent author.

## Abbreviations

anti- Tg: Thyroglobulin antibodies
anti-TPO: Anti-thyroid peroxidase antibodies
CO_2_ laser: Carbon dioxide laser
HIV: Human immunodeficiency virus
HSV: Herpes simplex virus
TSH: Thyroid- stimulating hormone
